# Independent value of PMOI on hCG day in predicting pregnancy outcomes in IVF/ICSI cycles

**DOI:** 10.3389/fendo.2023.1086998

**Published:** 2023-02-23

**Authors:** Xingyu Sun, Fei Yao, Chengliang Yin, Muzi Meng, Yunzhu Lan, Ming Yang, Chenyu Sun, Ling Liu

**Affiliations:** ^1^ Department of Gynecology, The Affiliated Traditional Chinese Medicine Hospital of Southwest Medical University, Luzhou, Sichuan, China; ^2^ Reproductive Medicine Center, the Affiliated Hospital of Southwest Medical University, Luzhou, China; ^3^ Faculty of Medicine, Macau University of Science and Technology, Macau, Macau SAR, China; ^4^ United Kingdom (UK) Program Site, American University of the Caribbean School of Medicine, Preston, United Kingdom; ^5^ Bronxcare Health System, New York City, NY, United States; ^6^ Obstetrics Department, The Fourth Affiliated Hospital, College of Medicine, Zhejiang University, Hefei, China; ^7^ Obstetrics Department, The First Dongguan Affiliated Hospital Of Guangdong Medical University, Dongguan, China; ^8^ Department of Thyroid and Breast Surgery, The Second Hospital of Anhui Medical University, Hefei, China

**Keywords:** progesterone to number of mature oocytes index, HCG day, pregnancy outcomes, IVF/ICSI cycle, prediction

## Abstract

**Objectives:**

The aim of this study was to determine whether, on the day of human chorionic gonadotropin (hCG) injection, the progesterone to number of mature oocytes index (PMOI) can be used alone or together with other parameters in a fresh embryo transfer *in vitro* fertilization (IVF)/intracytoplasmic sperm injection (ICSI) cycle to predict pregnancy outcome.

**Methods:**

This was a retrospective cohort study of all couples who underwent a clinical pregnancy and received a fresh IVE/ICSI cycle at a single large reproductive medical center between June 2019 and March 2022. The study involved a total of 1239 cycles. To analyze risk factors associated with pregnancy outcomes on the day of HCG injection, univariate and multivariate logistic regression analyses were used. The area under the curve (AUC) was determined, and PMOI and other factors were compared using receiver operating characteristic (ROC) curves.

**Results:**

The clinical pregnancy rate was significantly higher in group A (60.76%) than in the other groups (Group B: 52.92% and Group C:47.88%, respectively, p =0.0306). Univariate and multivariate logistic regression revealed that PMOI levels were significantly correlated with the probability of pregnancy outcome, independent of other risk factors. More importantly, PMOI levels independently predict the occurrence of pregnancy outcome, comparable to the model combining age. The optimal serum PMOI cutoff value for pregnancy outcome was 0.063 ug/L.

**Conclusion:**

Our results suggest that PMOI levels have an independent predictive value for pregnancy outcome in fresh IVF/ICSI cycles.

## Introduction

The term “infertility” refers to the inability to achieve a successful pregnancy after 12 months or more of appropriate, time-limited unprotected intercourse or therapeutic donor insemination. Nevertheless, early evaluation and treatment after six months may be reasonable for women over 35 years of age (1). A distressing fact about infertility is that the number of infertility patients is increasing every year, and infertility has become a major health problem, affecting 8%–15% of couples of reproductive age worldwide (2). Reassuringly, in recent years, assisted reproductive technology (ART), consisting of *in vitro* fertilization (IVF) and intracytoplasmic sperm injection (ICSI), has become an important treatment for many infertile women (3). However, despite recent advances in assisted reproductive technologies, success rates remain low, causing public socioeconomic distress regarding the health of individuals and women. Thus, achieving high pregnancy rates is our main challenge today with regard to assisted reproductive technologies (ART). Therefore, predicting pregnancy outcomes after assisted reproductive technologies has been a research hotspot, and more evidence is needed to help inform couples undergoing assisted reproduction, clinicians and policy makers. Currently, the factors that predict pregnancy outcomes on the day of HCG injection are not fully understood.

Progesterone (P) is essential before and during pregnancy as it plays a key role in supporting the endometrium and thus fetal survival (4). In the natural cycle, preovulatory P secretion facilitates the action of estrogen on the pituitary gland; the latter is a key factor in producing a mid-cycle luteinizing hormone (LH) peak. In addition, progesterone also stimulates a mid-cycle follicle-stimulating hormone (FSH) surge, which is important to support the expression of LH receptors in the granulosa layer (5, 6). Notably, the majority of circulating P (~95%) is produced in the follicle by granulosa cells (GCs) through the action of 3β-HSD catalyzing the conversion of pregnenolone (delta-4 pathway) under the influence of LH (7, 8). After ovulation, the corpus luteum is formed, and both the corpus luteum and GCs produce P in response to endogenous LH activity (9). In early pregnancy, human chorionic gonadotropin (hCG), secreted by syncytial trophoblasts, rescues the corpus luteum and maintains luteal function until the establishment of placental steroidogenesis (10). Therefore, elevated progesterone (PE) and its sustained levels are considered essential to be key in eliciting the endocrine signals responsible for initiating the endometrial receptive phase to embryo implantation (11, 12).

Nevertheless, it has been debated in the literature for over two decades that elevated serum progesterone (SP) is possible deleterious effects on the day of human chorionic gonadotropin (hCG) administration in relation to the outcome of assisted reproductive technology (ART) cycles (13, 14). Previous studies have reported that the decrease in clinical pregnancy rates (CPRs) was statistically significant (P<0.05), whereas no association was found in others (15, 16). Simultaneously, it is not widely accepted that serum progesterone (SP) has an adverse effect on cycle outcomes at a specific threshold level. Hence, serum progesterone (SP) cannot serve as a sole predictor of clinical pregnancy (CP). To our knowledge, SP level may correlate with the number of hormonally active follicles. In addition, the number of mature oocytes was a more objective parameter. As such it may be possible to use a new parameter of measurement: the progesterone to number of mature oocytes index (PMOI) to predict successful clinical pregnancy (CP) compared to SP levels alone.

Wu et al. showed that elevated progesterone on the day of hCG triggering was associated with a detrimental effect on live birth rate in low and intermediate ovarian responders, but not in high responders (17). It was a retrospective study with 2,351 patients receiving fresh assisted reproduction technology (ART) transfer cycles with GnRH agonist using a long or short protocol. Currently, the underlying mechanism of PE on IVF pregnancy outcome remains unclear, endometrial receptivity and embryo quality are two key factors to the success of implantation (18). Lu et al. has demonstrated PE influences endometrial receptivity during fresh ET cycles (19). It is possible that PE will promote the endometrium without affecting the embryo, which can lead to a dyssynchrony between the embryo and endometrium, which could lead to a decrease in the implantation rate, thereby reducing pregnancy rates. Furthermore, Sahar et al. also displayed that pregnancy rates were significantly lower on the day of hCG administration with progesterone thresholds above 1.5ng/mL compared to progesterone levels lower than 1.5ng/mL (20). Since progesterone (P) alone will not predict pregnancy outcome, various markers have been proposed to predict the outcome of assisted reproductive cycles more accurately.

As for strengths of PMOI, on the one hand, a correlation has been found between serum progesterone levels and the number of follicles with hormonal activity (21). It is possible for the number of follicles seen on ultrasound examination to vary between observers. On the other hand, it was suggested that the number of oocytes retrieved could be used as an objective parameter to calculate the progesterone to number of mature oocytes index (PMOI) (22, 23).

This aim of this study was to investigate the risk factors for pregnancy outcome in patients treated with fresh cycles of *in vitro* fertilization (IVF) or intracytoplasmic sperm injection (ICSI) and to elucidate the predictive power of PMOI level on the day of human chorionic gonadotropin (hCG) injection on pregnancy outcome based on retrospective data analysis. These results can help medical professionals take steps to minimize pregnancy failure and aid in decision-making for fresh embryo transfer cycle.

## Materials and methods

### Study population

**Figure 1:**
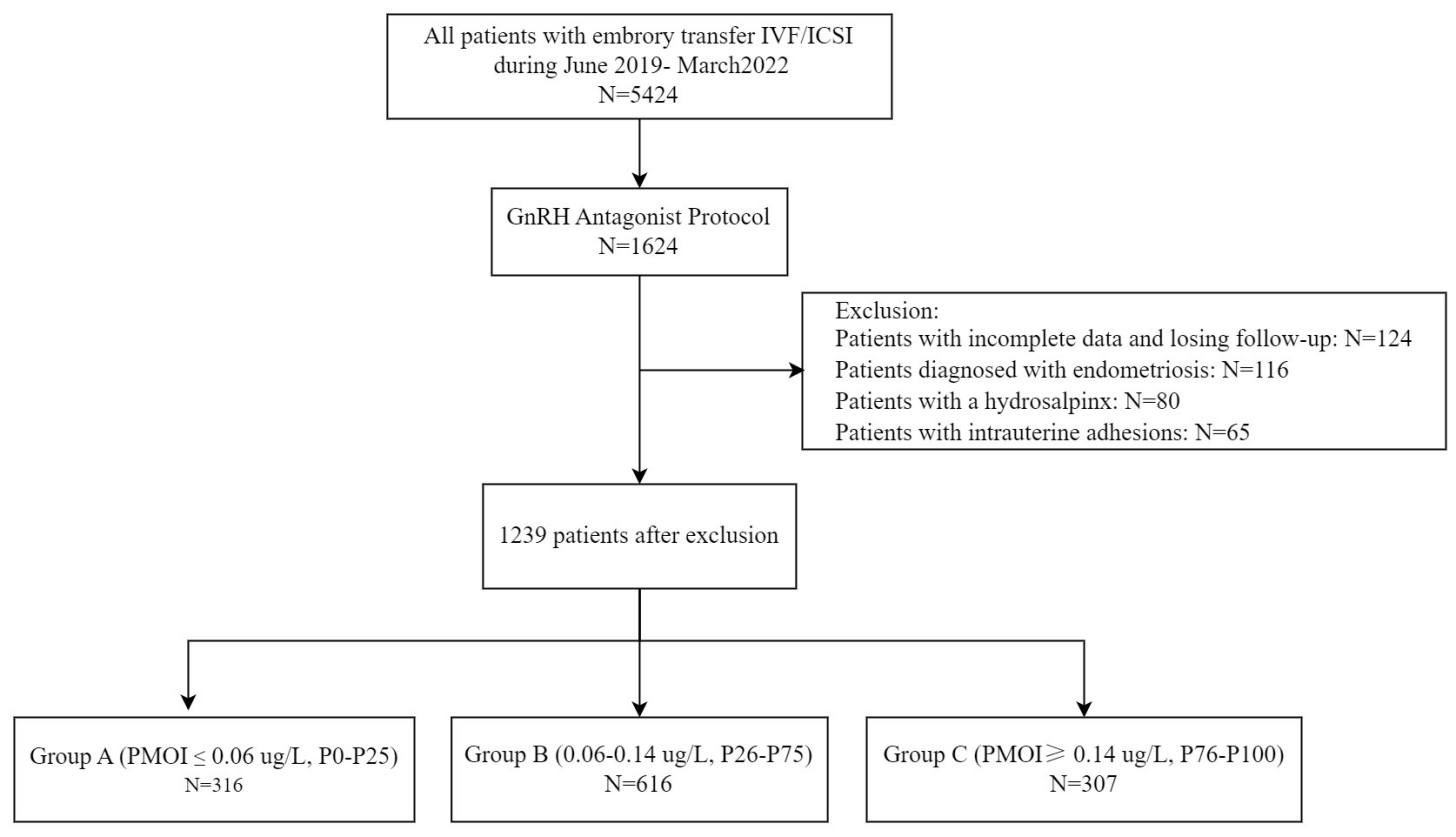
Flow chart of the research.

In the cross-sectional retrospective study, enrolled patients underwent IVF/ICSI cycles at the reproductive center of the Affiliated hospital of Southwest Medical University, located in Luzhou, China. Medical records of all patients treated by IVF/ICSI from June 2019 and March 2022 were screened. The criteria for inclusion and exclusion of patients from this study are listed below. The flow chart for patient selection is shown in **Figure 1**.

#### Inclusion criteria

(1) Women undergoing their first IVF/ICSI cycle for unexplained infertility, tubal infertility, stage I/II endometriosis, or a partner diagnosed with male-factor infertility were included in the study.
(2) Patients were treated with a gonadotropin-releasing hormone (GnRH) long-acting agonist regimen and received IVF/ICSI-ET.

#### Exclusion criteria

(1) Patients with incomplete data and loss to follow-up were excluded.
(2) Patients diagnosed with endometriosis prior to embryo transfer were excluded from the study.
(3) Patients with hydrosalpinx by hysterosalpingography (HSG) prior to embryo transfer were excluded.
(4) Patients with intrauterine adhesions diagnosed by hysterosalpingography (HSG) were excluded.
(5) Patients with no available embryos for transfer or canceled cycles for other reasons were excluded.

### GnRH antagonist protocol

All women underwent controlled ovarian stimulation (COS) with a GnRH antagonist fixed regimen. Bilateral antral follicles (10mm) were counted by transvaginal ultrasonography on the second day of the menstrual cycle, and women started COS treatment with gonadotrophins (Gonal-F, Merck Serono Europe Ltd or Puregon, N. V. Organon). The levels of serum progesterone (SP) were measured using an automated electrochemiluminescence immunoassay (Roche Diagnostics Elecsys Cortisol II assays and COBAS E801), and values were expressed in ng/ml. At our center, the starting dose of gonadotrophins was 150 IU/day for women aged ≤ 34 years, with BMI <24 kg/m^2^, 6≤AFC<15, and the dosage would be increased if the woman was older (age ≥ 35 years), heavier (BMI ≥ 24 kg/m2), or had poorer ovarian reserve AFC<6 or basal FSH>10 IU/L or AMH <1 ng/ml. Conversely, if the woman is lean (BMI < 19 kg/m2) or has a good ovarian reserve (AFC≥ 15 or AMH≥ 4 ng/ml), the dosage will be reduced. Dose adjustments were determined by the physician based on individual clinical experiences. GnRH antagonists (Cetrorelix, BaxterorGanirelix, N.V.Organon) were given daily starting on day 5 or 6 of stimulation. Human chorionic gonadotrophin (Chorionic Gonadotrophin for Injection, Livzon) was injected once there were at least three follicles >17 mm in diameter or at least two follicles >18 mm in diameter. Oocyte retrieval was performed under ultrasound guidance 34-36 hours after triggering.

### Grouping

Patients were divided into three concentration groups according to the percentage of PMOI levels on the day of hCG injection: Group A (PMOI ≤ 0.06 ug/L, P0-P25), Group B (0.06-0.14 ug/L, P26-P75), and Group C (PMOI≥ 0.14 ug/L, P76-P100). Clinical data, including age, infertility duration, BMI, and other relevant clinical data, were compared between the groups.

### Statistical analysis

Data analysis was performed using SPSS 23.0. Continuous variables were expressed as mean ± standard deviation (SD), and categorical variables were expressed as N (%). Univariate and multivariate logistic regression analyses were performed to assess risk factors associated with pregnancy outcomes. Receiver operating characteristic (ROC) curves were plotted, the area under the curve (AUC) was calculated, and the relationship between PMOI and other factors was compared. Optimal cutoff values were estimated by using the Youden index.

## Results

### Comparison of clinical data in each group

Patients were divided into three concentration groups according to the percentage of PMOI levels on the day of hCG injection: Group A (PMOI ≤ 0.06 ug/L, P0-P25), Group B (0.06-0.14 ug/L, P26-P75), and Group C (PMOI≥ 0.14 ug/L, P76-P100). There were significant differences in the general characteristics of the three groups, including AFC, FSH, FSH/LH, AMH, T, P, E2 on hCG day, number of eggs obtained, number of M_II_ oocytes, number of available embryos and clinical pregnancy rate. Group A had the highest AFC, FSH, FSH/LH, AMH, P, E2 on HCG day, number of eggs obtained, number of M_II_ oocytes, number of available embryos, and clinical pregnancy rate. **Table 1** summarizes these data and provides additional information.

**Table 1:** Comparison of clinical data in each group.

Parameters	Group A	Group B	Group C	*P value*
No. of cases	316	616	307	–
Age(year)	30.14 ± 4.36	31.06 ± 4.47	32.06 ± 5.14	0.765
Infertility duration	4.59 ± 3.48	4.56 ± 3.47	4.58 ± 3.53	0.991
BMI	23.34 ± 3.43	22.41 ± 3.24	22.32 ± 3.18	0.664
AFC	10.91 ± 4.41	10.12 ± 4.53	8.79 ± 4.18	<0.05
FSH	7.49 ± 2.16	8.15 ± 2.21	8.22 ± 1.99	<0.05
FSH/LH	2.79 ± 2.74	3.28 ± 3.54	3.29 ± 3.14	0.042
E2	53.63 ± 48.33	63.88 ± 79.04	66.32 ± 80.06	0.0589
AMH	5.24 ± 4.24	4.09 ± 3.50	2.89 ± 2.79	<0.05
PRL	12.75 ± 7.68	14.60 ± 15.86	14.65 ± 8.64	0.0766
T	45.90 ± 19.35	42.96 ± 19.90	46.56 ± 18.79	0.843
P	0.60 ± 0.42	0.65 ± 0.43	0.72 ± 0.38	0.0462
Endometrial thickness on transfer day	6.50 ± 2.42	6.63 ± 2.45	6.96 ± 2.54	0.0539
Total dosage of Gn used	2279.45 ± 743.23	2429.19 ± 712.90	2522.23 ± 805.24	0.324
Gn used duration	9.80 ± 2.34	9.84 ± 2.15	9.72 ± 2.64	0.849
E2 on HCG day	2810.39 ± 1278.89	2755.21 ± 1494.15	2027.26 ± 1002.33	< 0.05
No. of eggs obtained	11.29 ± 4.46	9.88 ± 4.23	6.67 ± 3.96	< 0.05
No.of MII oocytes	10.32 ± 4.27	8.98 ± 4.04	6.06 ± 3.78	< 0.05
No.of available embryos	4.12 ± 1.23	3.56 ± 1.25	2.54 ± 1.01	<0.001
No.of transferred embryos	1.69 ± 0.58	1.71 ± 0.48	1.53 ± 0.52	0.465
Clinical pregnancy rate	60.76(192/316)	52.92(326/616)	47.88(147/307)	0.0306

### Univariate and multivariate analyses of clinical factors

A univariate logistic regression analysis was performed, considering all factors that may have an impact on clinical pregnancy rates. The results showed that age (OR=0.772, P=0.027), available embryos (OR=1.287, P=0.034), and PMOI (OR=0.002, P=0.010) were all associated with clinical pregnancy rate (P<0.05). The results are shown in the logistic regression table. Later, independent factors such as age, available embryos, and PMOI were included together in a multivariate logistic regression model. These results showed that age and PMOI were independent risk factors for clinical pregnancy rate. Detailed results are shown in **Table 2**.

**Table 2:** Univariate and multivariate analyses of clinical factors.

Variables	Univariate analysis		Multivariate analysis	
	*P*	*OR (95%CI)*	*P*	OR (95%CI)
Age	0.027	0.772(0.644-0.942)	0.032	0.742(0.578-0.863)
E2 on the day of hCG injection	0.524	1.000(1.000-1.0000)	–	–
P on the day of hCG injection	0.267	0. 817(0.571-1.168)	–	–
No.of M_II_ oocytes	0.315	0.941(0.836-1.059)	–	–
No.of available embryos	0.034	1.287(1.057-1.363)	0.126	1.194(0.857-01.384)
No.of 2PN	0.604	1.070(0.828-1.383)	–	–
No.of transferred embryos	0.130	1.353(0.879-2.556)	–	–
PMOI on hCG injection day	0.010	0.002(0.000-0.227)	0.043	0.005(0.000-0.780)

### Predictive value of PMOI for pregnancy outcomes

**Figure 2:**
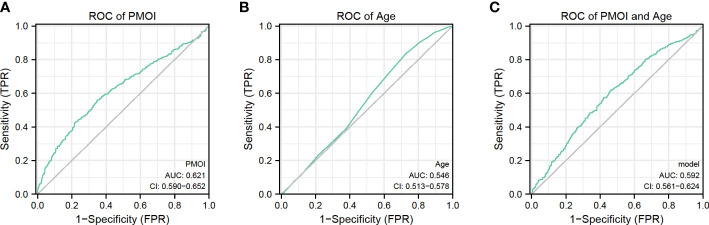
Predictive value of PMOI and age for pregnancy outcomes.

We performed a ROC curve analysis to explore the predictive value of PMOI levels and other risk factors for pregnancy outcome (**Figure 2**). The AUC for PMOI levels was 0.621ug/L (**Figure 2A**). The optimal PMOI threshold for predicting pregnancy outcome was 0.063, with a specificity of 79.2% and a sensitivity of 42.7%, according to the Youden index algorithm in the ROC curve. Meanwhile, the age-level AUC was 0.546 (**Figure 2B**). In the ROC curve, the optimal age threshold for predicting pregnancy outcome based on the Youden index algorithm was 34.5, with a specificity of 27.4% and a sensitivity of 83.5%. In addition, a prediction model combining PMOI and age was developed. The AUC of this model was 0.592 (95%CI:0.561–0.624). As for age, the AUC of the model increased from 0.546 to 0.592 (95% CI 0.561-0.624; P < 0.05) after including PMOI in age (**Figures 2C**, **Table 3**). Therefore, we can naturally conclude that PMOI levels can be used as an independent predictor of clinical pregnancy rate. Detailed results are shown in **Table 3** and **Figure 2**.

**Table 3:** Accuracy of different variables and model in IVF/ICSI to predict clinical pregnancy rate.

Parameters	AUC	95% CI	P valve	Best threshold	Specificity(%)	Sensitivity(%)
PMOI	62.1	0.590-0.652	<0.05	0.063	78.2	42.7
Age	0.546	0.513-0.578	<0.05	34.5	27.4	83.5
Model	0.592	0.561-0.624	<0.05	0.160	53.7	62.1

AUC, area under the curve; CI, confidence interval; NPV, negative predictive valve; PPV, positive predictive valve.

Model included PMOI and Age.

## Discussion

In this study, we demonstrated that in fresh IVF/ICSI cycles, PMOI levels were significantly associated with the risk of pregnancy outcome on the day of hCG injection, independent of other risk factors, including age, E2 on the day of hCG injection, P on the day of hCG injection, number of M_II_ oocytes, number of available embryos, 2PN count, and number of transferred embryos. In addition, PMOI showed a more significant AUC than age in predicting pregnancy outcome on the day of hCG injection. More importantly, our findings suggest that PMOI has a more significant predictive value than the model including PMOI and age.

Progesterone (P) is known to perform an important physiological function during the menstrual cycle and pregnancy (24). The use of late follicular P levels to predict pregnancy outcome in assisted reproductive therapy (ART) remains controversial. A recent study showed that elevated P levels on the day of human chorionic gonadotropin (HCG) administration had a negative impact on live birth rate and were associated with high miscarriage rates. However, the adverse effects of high P levels during pregnancy were not associated with endometrial receptivity (25). A different study reported that low P levels (≤ 0.5 ng/ml) on the day of hCG administration were associated with a low live birth rate (LBR) (26). Several studies have reported that prematurely elevated P levels on the day of hCG administration were negatively associated with IVF outcomes in cycles with gonadotropin-releasing hormone (GnRH). Sangisapu et al. showed no predictive association with either IVF outcomes or progesterone levels in their study, a single-center retrospective cohort study conducted on 306 fresh IVF cycles of normozoospermic semen samples and COS by long protocol with GnRH agonists followed by hCG trigger from 2016 to 2018 (27). In addition, different P thresholds were used in different studies. Deng et al. demonstrated a negative correlation and saturation effect between serum progesterone and first pregnancy outcome. When progesterone was <90.62 nmol/L, a 1 nmol/L increase in serum progesterone was associated with 3% reduction in the risk of miscarriage (OR: 0.97, 95% CI: 0.95-0.98) (28). To address these deficiencies, we conducted a retrospective cohort study of PMOI levels to predict pregnancy outcomes in patients.

In this study, the lowest clinical pregnancy rate (60.76%) was found in group C among groups A, B, and C. This may be partly due to the identified increase in serum progesterone levels. Our findings are supported by a number of studies that report a significant decrease in pregnancy rates due to increased progesterone levels (29–31). Sahar et al. demonstrated that progesterone levels > 1 ng/mL on the day of hCG administration decreased HOXA10 expression so that endometrial reception during the implantation period was already impaired (20). Various factors of clinical pregnancy on the day of hCG were examined by both univariate and multivariate analyses. The results showed that PMOI (OR: 0.005, 95%CI: 0.000-0.780, P=0.043) was an independent risk factor for pregnancy outcome. In our study, the risk of obtaining the number of eggs, the number of M_II_ oocytes, and the number of available embryos decreased as PMOI increased. Our study suggests that PMOI may have a negative impact on pregnancy outcome. However, this study was a single-centre study with a small overall sample size, so whether PMOI on hCG day is associated with poor pregnancy outcomes remains to be further confirmed in a large-scale clinical studies. Grin et al. noted that higher serum progesterone levels are associated with follicular counts and that higher P affects endometrial receptivity, as well as oocyte and embryo quality (32). Their study showed POI (ration of number of oocytes aspirated) was inversely related to CP (clinical pregnancy) with an adjusted OR of 0.063 (95% CI: 0.016-0.249, p <.001). POI is a simple predictor of IVF-ET cycle outcome, and it can advocate a limit beyond which embryo transfer should be reconsidered. Simon et al. showed that PMOI recorded in the same patient in a single attempt was similar and partially correlated with basal FSH, anti-Müllerian hormone, antral follicle count, and OSI (33). In the current study, univariate and multivariate regression analyses showed that age and PMOI on the day of hCG injection were independent prognostic factors affecting the outcome of IVF/ICSI-ET pregnancies. Also, in the current study, the results of the ROC curve showed that the threshold value of PMOI on the day of hCG injection to predict pregnancy outcome was 0.063. The sensitivity was 42.7%, and the specificity was 78.2%. The AUC obtained was 62.1 (P<0.05), which shows that PMOI on the day of hCG has some value in predicting pregnancy outcome.

The PMOI seems to be elevated mainly in patients with low ovarian reserves and low ovarian response as evaluated by ovarian sensitivity index (OSI). Indeed, PMOI seemed to be reproducible from one attempt to another in the same patient and is related to low AMH and AFC levels. As a matter of fact, in poor responders, the higher administrated FSH doses result in a higher FSH dose to recruited follicle ratio, probably leading to a higher follicular fluid progesterone concentration. Therefore, the freeze-all strategy and reducing the FSH doses are probably the right way to avoid or limit oocyte damage. Due to the respective parts of the effect on the endometrium and on embryo developmental ability are difficult to discriminate, the use of PMOI could help to identify an oocyte effect rather than an endometrial one. In brief, the PMOI seemed to be more predictive of IVF outcomes than blood progesterone levels.

The underlying mechanism of the premature rise of progesterone (P) on hCG day is not fully understood and may be related to the following factors. First, granulosa cells have abundant receptors on their surface, such as follicle-stimulating hormone (FSH), luteinizing hormone (LH), and estrogen receptors (34). High-dose FSH increase the sensitivity of FSH-incuded LH receptors in granulosa cells, leading to increased LH levels (26). Secondly, ovarian stimulation leads to maturation of multiple follicles and therefore supraphysiological concentrations of progesterone in the early luteal phase (35). Third, premature rise in progesterone (P) is associated with a low ovarian reserve and poor ovarian response (36). Fourth, patients with poor ovarian response are more likely to have an elevated estradiol/testosterone ratio (32). Patients who obtain fewer than five eggs may see more elevated POI (36). This was confirmed by the present study. We found that the highest FSH levels were in group A (PMOI ≤ 0.06 ug/L). While the results for groups A to C were 7.49 ± 2.16, 8.15 ± 2.21, and 8.22 ± 1.99, respectively. In addition, the lowest AMH and AFC were found in group A (PMOI ≤ 0.06 ug/L). These differences showed statistical significance. This present study suggests that patients with low ovarian response are more likely to have elevated PMOI on the day of hCG injection.

The strength of the present study is based on a specific population living in Southwest China, suitable for economically undeveloped areas, but with an increasing trend of infertility patients. In addition, these measurements were performed in the same laboratory using the same equipment. This greatly reduces the variability caused by laboratory testing. Our study, however, has some limitations. On the one hand, this was a retrospective study. On the other hand, the current PMOI predictive values in this study showed an average performance in predicting pregnancy outcomes, with a specification of 78.2% and a sensitivity of 42.7%. Therefore, there is a need to improve the ability to predict pregnancy outcomes in fresh IVF/ICSI cycles beyond the current capabilities.

In conclusion, the present study shows that PMOI is an independent and meaningful predictor of pregnancy outcome on the day of hCG injection in fresh IVF/ICSI cycles and that combining age and PMOI levels does not improve the prediction effect.

## Data availability statement

The original contributions presented in the study are included in the article/supplementary material. Further inquiries can be directed to the corresponding authors.

## Ethics statement

Ethics approval for this study was obtained from The Affiliated Hospital Of Southwest Medical University Ethics Committee (No.KY2022312). Informed consent from study participants was not required.

## Author contributions

LL and CS: Conceptualization. XS and FY: Data curation, Writing-Original draft preparation. MM, CY, MY, and YL: revising the manuscript critically for important intellectual content. All authors contributed to the article and approved the submitted version.
